# The Revived Interest in Ageusia Research during the COVID-19 Pandemic: A Bibliometric Analysis

**DOI:** 10.3390/life13041062

**Published:** 2023-04-21

**Authors:** Andy Wai Kan Yeung

**Affiliations:** Oral and Maxillofacial Radiology, Applied Oral Sciences and Community Dental Care, Faculty of Dentistry, University of Hong Kong, Hong Kong, China; ndyeung@hku.hk

**Keywords:** ageusia, COVID-19, bibliometric, VOSviewer, Bradford’s law

## Abstract

The evolution of ageusia research literature has yet to be investigated. This bibliometric study analyzed the entire ageusia research literature indexed in Web of Science, to reveal its growth and the most productive entities in terms of authors, institutions, countries, journals, and journal categories. In addition, this study aimed to identify medical conditions (and their treatments) that were frequently associated with ageusia. On 7 March 2022, the Web of Science Core Collection database was accessed with the following search query: TS = (ageusia OR “taste loss” OR “loss of taste” OR “loss of gustat*” OR “gustatory loss”). The search identified publications mentioning these terms in their title, abstract, or keywords. No additional filters were placed on publication year, language, etc. The basic publication and citation counts were extracted from the in-built functions of the database. The complete record of the publications was exported into VOSviewer, a bibliometric software for visualizations. The search yielded 1170 publications. The cumulative publication and citation counts of the ageusia research sharply increased in 2020. The most productive author was Professor Thomas Hummel from Technische Universität Dresden. Ageusia research had heavy contributions from the United States, Italy, the United Kingdom, Germany, and India. The top 5 most productive journals mainly belonged to the otorhinolaryngology and medicine categories. The medical conditions frequently investigated in ageusia research included COVID-19, cancers (head and neck, and advanced basal cell), Guillain-Barré syndrome, neurodegenerative diseases, diabetes, and Sjogren’s syndrome. This study could act as a begvinner’s guide for (1) clinicians who are not familiar with ageusia so that they might better understand which scenarios they need to be more aware of since ageusia could be a co-morbidity of a patient’s underlying disease, and (2) for those who wish to search for relevant authors and journals for suitable publications related to the topic.

## 1. Introduction

One common symptom experienced by patients with COVID-19 is taste dysfunction. A recent meta-analysis revealed that 48.1% of patients with COVID-19 had taste disorders, with 28.0% of patients experiencing ageusia [1]. By definition, ageusia is the absence of any taste sensation [2]. These numbers must be much higher than those from the general population, as a retrospective study on 1176 patients attending a chemosensory clinic reported only 0.4% of patients with ageusia [3].

The exact pathogenesis of COVID-19-related ageusia remained to be elucidated. It was identified that the angiotensin-converting enzyme 2 (ACE2) was the cellular receptor for the SARS-CoV-2 virus [4]. Meanwhile, there are abundant ACE2 receptors on the oral mucous membrane especially on the tongue [5]. It was well-known that prolonged use of ACE inhibitors such as some common antihypertensive drugs tended to associate with altered taste perception [6]. Hence, it was proposed that the binding of the SARS-CoV-2 virus to ACE2 might contribute to taste alteration and even taste loss [7] after the virus infected the taste cells leading to inflammation and cell death [8]. Another possibility could be the binding of the SARS-CoV-2 virus to another site called the sialic acid receptor on the taste buds and the subsequent acceleration of the gustatory molecules to degrade, which could lead to attenuated taste [9,10]. The third plausible mechanism could be through a direct infection and damage to the cranial nerves responsible for taste sensation, such as the facial nerve, the glossopharyngeal nerve, and the vagus nerve [11]. Some studies reported that patients with COVID-19 suffered from sour and salty taste suppression more severely than bitter and sweet tastes, and suggested that the differences were due to the former two taste qualities being transmitted by ion channels whereas the latter were by transduction via G-protein-coupled receptors [12,13]. Regardless of the pathogenesis, ageusia could have serious implications for the well-being of the affected patients, as a recent study showed that COVID-19-related ageusia might increase the risk of depression and suicidal ideation by 30% [14]. It was suggested that the loss of taste sensation could even affect sexual arousal with reduced sensory stimulation during sexual activities, which might contribute to COVID-19-related transitory erectile dysfunction [15]. Overall, the severity of taste dysfunction was positively correlated to the reduction in quality of life in terms of mental components [16]. However, initial findings showed reassuring results that such taste dysfunction was mostly transient that would resolve in the majority of cases after 6 months of post-COVID-19 infection [16]. 

Meanwhile, there are other medical conditions associated with ageusia. Guillain-Barré syndrome is a syndrome for which the peripheral nervous system experiences idiopathic damage from the immune system, which, in rare cases, may cause ageusia when the chorda tympani is damaged [17]. Sjogren’s syndrome is an autoimmune disease that primarily affects the lacrimal and salivary glands leading to dryness (such as the eyes and oral cavity), pain, and fatigue [18]. With salivary dysfunction, it was found that many patients with Sjogren’s syndrome suffered from dysgeusia and even ageusia, affecting their oral health-related quality of life [19,20]. Impaired taste sensation or even ageusia was also found among patients with diabetes [21]. The exact cause was unknown, but possible reasons included inherent or acquired taste receptor defect, peripheral neuropathy, and altered cerebral processing of taste [21].

With the background of the COVID-19 pandemic, ageusia research should have gained much popularity in the past couple of years. However, there has yet to be a bibliometric analysis to reveal the latest developments in the ageusia research literature. A recent study analyzed the literature on taste and smell dysfunction in COVID-19 [22]. This study, therefore, aimed to investigate the ageusia research literature without a publication year limit, to reveal its growth and the most productive entities in terms of authors, institutions, countries, journals, and journal categories. In addition, this study aimed to identify medical conditions (and their treatments) that were frequently associated with ageusia, so that clinicians not familiar with ageusia could better understand which scenarios they should become more aware of regarding the possibility of ageusia being a co-morbidity of a patient’s underlying disease.

## 2. Materials and Methods

On 7 March 2022, the online database Web of Science Core Collection (According to the subscription plan of the author’s affiliation, Web of Science Core Collection refers to: Science Citation Index Expanded (1970–present), Social Sciences Citation Index (1956–present), Arts & Humanities Citation Index (1975–present), Conference Proceedings Citations Index—Science (2009–present), Conference Proceedings Citation Index—Social Science & Humanities (2009–present), Emerging Sources Citation Index (2005–present)) was accessed with the following search query: TS = (ageusia OR “taste loss” OR “loss of taste” OR “loss of gustat*” OR “gustatory loss”). The exact specifications of sub-datasets and coverage timespans were provided as a practice advocated by [23]. The search identified publications mentioning these terms in their title, abstract, or keywords. No additional filters were placed on publication year, language, etc. The search yielded 1170 publications.

The basic publication and citation counts were extracted from the in-built functions of the database. The counts from England, Scotland, Northern Ireland, and Wales were combined to represent the United Kingdom. The complete record of the publications was exported into VOSviewer [24], a bibliometric software, for visualizations of a term map. The default parameters were used. The term map illustrated the recurring terms from the title and abstract of the publications. For clarity, the map only showed terms that occurred in at least 1% (n = 12) of the publications. Each term was represented by a circle, for which the size reflected the publication count, the color reflected the citations per publication (CPP), and the distance with other terms reflected their co-occurrence. In addition, the top 10 most cited publications were identified and the context of their citations (supporting, mentioning, or contrasting) were identified from another online database, Scite [25]. Through machine learning based on 25 million full-text academic articles, Scite is able to categorize citations based on the semantic context which enables researchers to conduct such analysis in a more objective/automated manner to reduce manual bias [25].

## 3. Results

**Figure 1:**
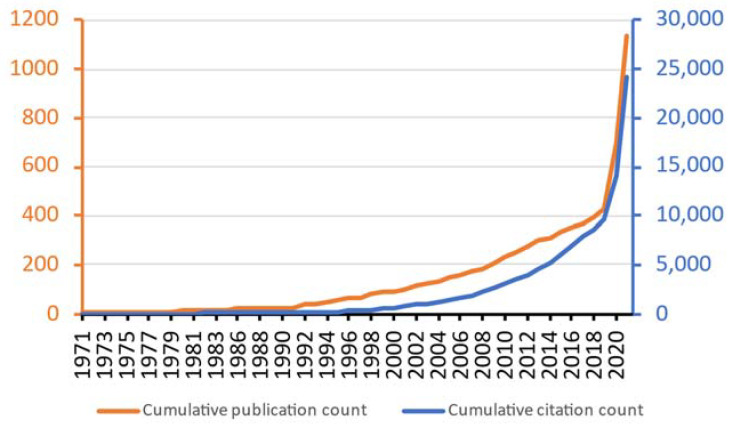
Cumulative publication and citation counts in ageusia research.

Figure 1 shows the cumulative publication and citation counts of the ageusia research. Both metrics sharply increased in the year 2020. The annual publication count actually rose from 31 (the year 2019) to 270 (2020) and 439 (2021), and the annual citation count rose from 1059 (the year 2019) to 4271 (2020) and 10,117 (2021). Three-quarters of the publications were original articles (n = 876, CPP = 21.8) whereas the rest were mainly review papers (n = 167, CPP = 30.0). Table 1 lists the top 5 most productive authors, institutions, countries, journals, and journal categories. The most productive author was Professor Thomas Hummel from Technische Universität Dresden, whose research interests included loss of gustatory and olfactory sensations in different clinical groups such as patients with stroke [26], Parkinson’s disease [27], during radiochemotherapy [28], and COVID-19 [29]. Technische Universität Dresden was also among the top 5 most productive institutions together with two institutions from the United States, and one each from the United Kingdom and France. Meanwhile, the most productive countries were led by the United States, followed by Italy. The top 5 most productive journals mainly belonged to the otorhinolaryngology category, but medicine journals also had a heavy contribution together, such as Lancet (n = 13, ranked 7th), Cureus (n = 10, ranked 10th), and BMJ Open (n = 7, ranked 20th). Bradford’s law of scattering stated that the publications within a research field should be concentrated in core journals that accounted for 1/3 of the total publications, and if publications were equally divided into three portions, then the number of journals accounted for in each portion should be in the ratio of 1:n:n^2^ [30,31]. In this case, there were 51 core journals that collectively published 390 publications (1/3 of 1170). The 2nd and 3rd 1/3 of the publications were published in 214 and 396 journals, respectively. In other words, the ratio was 1:4.2:7.8, deviating from the expected ratio of 1:4.2:17.6 derived from the law.

**Figure 2:**
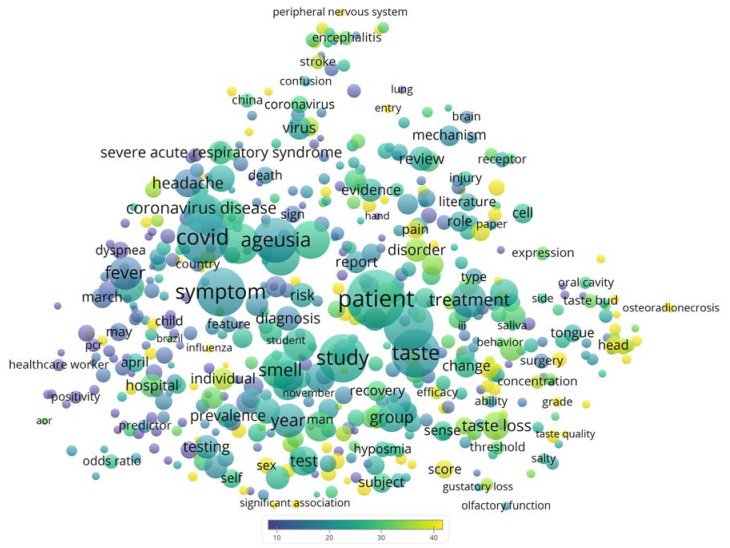
Term map showing recurring terms from titles and abstracts in ageusia publications.

COVID-19 and related terms were among the most recurring terms shown in Figure 2, such as COVID (n = 588, CPP = 20.1), coronavirus disease (n = 205, CPP = 26.2), and the previous coronavirus pandemic, severe acute respiratory syndrome (SARS, n = 124, CPP = 28.0). Meanwhile, the top 15 author keywords are listed in Table 2. Ageusia publications were often related to COVID-19, radiotherapy, head and neck cancer, children, and other taste and smell disorders. To reveal more diseases/treatments associated with taste loss, the authors’ keywords were further examined and relevant terms are listed in Table 3. They could be roughly grouped into cancers (head and neck, and advanced basal cell) and their associated therapy, Guillain-Barré syndrome, neurodegenerative diseases, diabetes, and Sjogren’s syndrome.

Finally, the top 10 most cited publications are listed in Table 4. Seven were related to COVID-19. Two were about cancer or cancer-related cases. The remaining study was a 7-year, single-center study with 750 patients. The ratio of supporting citations for these publications ranged from 0.5–8.4%. All these publications received more supporting citations than contrasting ones, whereas neutral (mentioning) citations formed the majority. The ratio of supporting to contrasting citations ranged from 2.9:1 to 12.5:1.

## 4. Discussion

This bibliometric study revealed that ageusia research was “revived” during the COVID-19 pandemic. Many research reports have been published over the last 2 years, with heavy contributions from the United States, Italy, the United Kingdom, Germany, and India. This was slightly different from the basic taste literature, for which the United States, Japan, Germany, and the United Kingdom were among the most productive countries [42]; and from the taste neuroscience literature, for which the top 5 were the United States, Germany, the United Kingdom, the Netherlands, and Japan [43]. A similarity shared among the three literature sets was that Chemical Senses and PLOS One were among the most productive journals, indicating their high relevance to food and taste scientists as well as clinicians. Notwithstanding, ageusia has undoubtedly attracted global research interest.

The exact mechanism for the SARS-CoV-2 virus to cause ageusia remains to be elucidated. It was suggested that the virus might occupy the binding sites of sialic acid on the taste buds to speed up the degradation of tastants [7]. Meanwhile, by examining author keywords, several other groups of diseases/treatments could be identified. First, head and neck cancer and its radiotherapy treatment might directly damage salivary glands, chorda tympani nerve, and taste receptors and lead to taste loss [36,44]. Meanwhile, hedgehog pathway inhibitors used to treat advanced basal cell carcinomas, such as vismodegib and sonidegib, could lead to transient loss of taste buds in fungiform and circumvallate papillae, as their differentiation and maintenance were regulated by hedgehog pathway signaling [45,46]. Second, Guillain-Barré syndrome, an autoimmune disorder that could possibly be associated with COVID-19 and its vaccine [47,48], might cause demyelination of chorda tympani and cause taste loss [49,50]. Third, neurodegenerative diseases such as Parkinson’s disease, multiple sclerosis, and amyotrophic lateral sclerosis could also be associated with ageusia, though the mechanisms were less clear. It was suggested that patients with Parkinson’s disease showed dysregulated taste receptor genes and possibly damaged glossopharyngeal nerves [51]. Meanwhile, patients with multiple sclerosis had lower taste identification scores if they had larger lesion volumes or a larger number of lesions in their brains [52]. For patients with amyotrophic lateral sclerosis, it was reported that taste perception was diminished at the fungiform and circumvallate papillae at the tongue and its medication riluzole might damage the chorda tympani [53]. These pieces of evidence suggested that neurodegenerative diseases could cause neural deficits centrally or peripherally leading to taste impairment and ageusia. Indeed, the cerebral processing of taste stimuli was altered in patients with taste loss compared to healthy controls, with a heightened signal from the insula and anterior cingulate but a dampened signal from the postcentral gyrus [54]. Four, ageusia was revealed in patients with diabetes [21,55] and obesity [55]. The exact mechanism was unclear, but angiotensin-converting enzyme inhibitors commonly prescribed to patients with diabetes as well as hypertension could make patients feel a lingering metallic, bitter, or sweet taste [56]. It should be noted that the anti-platelet medication clopidogrel was also well-known for its side-effect of ageusia that could be reversed upon treatment discontinuation [57]. Readers are referred to [56] for an extensive review of the diseases and therapeutic agents that cause taste dysfunction and ageusia.

The top 10 most cited publications had much more supporting citations than contrasting ones, in the ratio of 2.9:1 to 12.5:1. This ratio was consistent with other literature sets, such as scientific publications referenced in Wikipedia entries (8.8:1) and those indexed by Web of Science in general (6.8:1) [58]. Of course, most citations were neutral mentioning. These initial findings collectively suggested that the scientific literature in general seemed to be constructive without frequent arguments or rebuttals, for which the latter perhaps should be encouraged to allow a more balanced account of research findings and advancement. Meanwhile, this study found that the distribution of publications among journals did not follow Bradford’s law. This finding was coherent with recent studies for other literature sets, such as taste neuroscience, general neuroscience, ethnopharmacology, nutraceuticals, and systematic reviews for pneumonia [43,59,60,61,62]. Perhaps during this era of digital publishing, with so many new journals becoming available, bibliometricians should revisit Bradford’s law and evaluate if such tripartition of research output into 1:n:n^2^ journals is still valid for the majority of the literature sets and whether the core journals should still be those accounting for 1/3 of total publications.

Below, the top 10 most cited publications are discussed in further detail: 

The most cited publication was a multicenter cross-sectional study [32]. The study recruited 417 patients with COVID-19 who had mild-to-moderate symptoms from 12 European hospitals. The most prevalent symptoms were cough, myalgia, appetite loss, facial pain, and nasal obstruction. They found that 88.8% of recruited patients had some form of taste disorders including ageusia, and 72.8% of patients had constant and unchanged taste disorders on the days during treatment.

The second most cited publication was a review paper on the neurological associations of COVID-19 [33]. In the beginning, it listed the provisional definitions of confirmed, probable, and possible cases of numerous COVID-19-associated neurological conditions. For instance, to be a confirmed case of SARS-CoV-2 meningitis, encephalitis, myelitis, or central nervous system (CNS) vasculitis, one should (1) be able to detect the virus in the cerebrospinal fluid or brain tissue or have evidence of the virus-specific intrathecal antibody, and (2) find no other explanatory pathogen or cause. For acute disseminated encephalomyelitis associated with SARS-CoV-2 infection, Guillain-Barré syndrome, and other acute neuropathies associated with SARS-CoV-2 infection, in order to show a probable association, one needs to (1) have the neurological disease onset within 6 weeks of COVID-19 acute infection, (2) detect the virus RNA in any sample or show antibody evidence of acute COVID-19 infection, and (3) have no evidence of other commonly associated causes. Afterward, the paper summarized the findings from the existing literature on the prevalence, clinical and radiographic manifestations of COVID-19-associated neurological diseases, their pathophysiology, and relevant clinical, imaging, and lab investigations.

The third most cited publication was a clinical study on chemosensory evaluation published back in 1991 [34]. The study recruited 750 patients from a university smell and taste center and administered seven chemosensory tests to the patients after obtaining their background information through a questionnaire. An additional smell retest was administered to 306 patients during recall visits ranging from 5 months to 6.4 years. The results found that 3.1% of the recruited patients had ageusia. One-third of these patients with ageusia were due to iatrogenic reasons such as medication-induced, dental procedures, nasal operation, and radiation therapy. The rest were due to head trauma, upper respiratory infection/cold, idiopathic, and toxic chemical exposure. There were a few cases classified due to idiopathic reasons. While the exact reasons were not identified, differential diagnosis could include vitamin B12 deficiency and myeloproliferative disorder. It should be noted that there could be other iatrogenic causes of ageusia as reported from other studies [63,64], such as tonsillectomy, laryngoscopy, and middle-ear surgery (which may injure the chorda tympani).

The fourth most cited publication was a cross-sectional study that attempted to associate patients’ COVID-19 symptoms self-reported to a smartphone app and their actual results from the reverse transcription polymerase chain reaction (RT-PCR) diagnostic test [35]. Based on 18,401 participants, the study found that participants with a positive RT-PCR test result for COVID-19 had a much higher ratio of having loss of taste and smell than their counterparts with a negative RT-PCR test result (odds ratio being 6.7). The authors concluded that loss of taste and smell was a crucial symptom of COVID-19 and should be included as part of the routine screening for COVID-19.

The fifth most cited publication was a review paper on the consequences of head and neck radiotherapy [36]. One common consequence is taste loss, which is mainly caused by direct damage to the taste buds and/or their innervations due to radiation. However, the review also mentioned that taste loss is usually transient and may be able to return to normal levels 1–5 years after radiotherapy, depending on the radiation dose and the adaptational ability of the patient.

The sixth most cited publication was a randomized clinical trial that tested the efficacy of the medication vismodegib in treating basal cell carcinomas in 41 patients [37]. Results found that patients taking this medication routinely suffered from complications including taste loss, though it could effectively reduce the carcinoma tumor burden and block new growth.

The seventh most cited publication was a case-control study with 60 patients with COVID-19 and 60 age- and sex-matched controls without COVID-19 [38]. In the control group, no participants complained about taste loss. In the COVID-19 group, 17% of patients suffered from both taste and smell loss, whereas another 7% of patients suffered from taste loss only, indicating that chemosensory dysfunction was a symptom of COVID-19. 

The eighth most cited publication was a case report with two patients with COVID-19, one concurrently with Miller Fisher Syndrome and the other with polyneuritis cranialis [39]. Though these comorbidities seemed not to relate to ageusia, these patients still suffered from ageusia due to COVID-19 itself, again highlighting the relevance of it as an obvious symptom detected by patients with COVID-19.

The ninth and tenth most cited publication was a review paper on the neurologic manifestations of COVID-19 [40] and a letter to the editor that commented on the common findings from patients with COVID-19 [41]. Both publications reported that ageusia and anosmia were commonly reported symptoms by patients with COVID-19.

From the above summary of the 10 most cited publications, it can be observed that many of them concern ageusia in patients with COVID-19. Traditional topics about ageusia, such as its prevalence in the normal population, its relationship with head and neck radiotherapy, and induction by medication, were only covered by 3 out of the 10 papers. This suggests that research attention to ageusia was revived by its association with COVID-19. It is hoped that such attention could be sustained in the future, with more resources and manpower directed into the ageusia research field to further refine the understanding of its pathophysiology, diagnostic, and management methods, both related and unrelated to COVID-19. It should be noted that taste sensation is one of the five basic senses and its disturbance or even complete loss can severely affect a person’s quality of life. Moreover, it should also be noted that many recurring keywords listed in Table 2 were not included in the original search query that focused solely on ageusia and its variants. For example, dysgeusia, anosmia, and hyposmia were recurring keywords despite the fact that they were not included in the original search query. This suggests that patients with ageusia often came with comorbidities related to smell, or patients with ageusia were often investigated together along with patients with other chemosensory disorders.

Among the top 5 journals with the most publications, the Indian Journal of Otolaryngology and Head & Neck Surgery had a particularly lower CPP value. In actual fact, most of its ageusia publications were related to COVID-19, and recent findings suggested that publications related to COVID-19 generally received many citations compared to non-COVID-19 counterparts [65,66]. Therefore, the author speculated that the low CPP value of this journal could be explained by the fact that it was the only journal without an impact factor among the 5 listed in Table 1, so it had a citation bias against it.

The sudden increase in the publication count around the year 1990 could be generally explained by the fact that the Web of Science Core Collection became more comprehensive in indexing the abstract, author keywords, and keywords plus information [67]. It was found that many papers published before 1991 lack such information in the Web of Science Core Collection, hence the “hit rate” was lower before 1991 [68]. Meanwhile, the increase in the publication count around the year 2020 could also be generally explained by the publications of COVID-19-related papers [69].

This study had some limitations. Readers should be aware that this study relied on a single database, the Web of Science Core Collection, with a customized subscription. Publications not indexed by it would be missed in this study. For example, some publications indexed in Scopus and some preprints would be missed. Scopus has been used by many bibliometric studies as it covers a wider journal range [70]. Meanwhile, preprint platforms were important dissemination platforms during the COVID-19 pandemic, which posted over 6700 COVID-19-related preprints from January to April 2020 [71]. In addition, there was a possibility that some papers were no longer cited due to “obliteration by incorporation” [72,73], meaning that their findings have become so well-known that there is no longer a perceived need to cite the original references. As a result, the citation count might not fully reflect the attention/popularity of certain papers and topics. Moreover, the analysis of ageusia alone is less comprehensive than the analysis of all taste/smell dysfunction.

## 5. Conclusions

In conclusion, this bibliometric study revealed that ageusia research has gained much attention during the COVID-19 pandemic. Ageusia research had heavy contributions from the United States, Italy, the United Kingdom, Germany, and India. The top 5 most productive journals mainly belonged to the otorhinolaryngology category, but medicine journals also had a heavy contribution together. The medical conditions frequently investigated in ageusia research included cancers (head and neck, and advanced basal cell), Guillain-Barré syndrome, neurodegenerative diseases, diabetes, and Sjogren’s syndrome.

## Figures and Tables

**Table 1:** The top 5 most productive authors, institutions, countries, journals, and journal categories.

	Number of Publications (% of 1170)	Citations per Publication (CPP)
**Author**		
Hummel, Thomas	21 (1.8)	31.3
Vaira, Luigi Angelo	18 (1.5)	57.8
De Riu, Giacomo	16 (1.4)	63.2
Salzano, Giovanni	13 (1.1)	77.5
Doty, Richard L.	11 (0.9)	117.4
Hopkins, Claire	11 (0.9)	36.0
**Institution**		
University of London	40 (3.4)	55.3
University of California system	29 (2.5)	40.9
Harvard University	24 (2.1)	38.3
Technische Universität Dresden	23 (2.0)	31.3
Universite de Paris	22 (1.9)	83.7
**Country/region**		
United States	313 (26.8)	31.5
Italy	117 (10.0)	36.8
United Kingdom	96 (8.2)	39.5
Germany	91 (7.8)	23.5
India	73 (6.2)	19.3
**Journal**		
Laryngoscope	22 (1.9)	35.8
European Archives of Oto-Rhino-Laryngology	21 (1.8)	80.1
Indian Journal of Otolaryngology and Head & Neck Surgery	20 (1.7)	3.1
PLOS One	17 (1.5)	14.5
Chemical Senses	15 (1.3)	32.1
Journal category		
Medicine General Internal	153 (13.1)	17.0
Otorhinolaryngology	147 (12.6)	39.4
Neurosciences	119 (10.2)	21.6
Clinical Neurology	114 (9.7)	35.7
Surgery	64 (5.5)	29.1

**Table 2:** Top 15 author keywords in ageusia research.

Keyword	Number of Publications (% of 1170)	Citations per Publication (CPP)
COVID-19	434 (37.1)	15.5
SARS-CoV-2	211 (18.0)	18.7
Ageusia	155 (13.2)	17.0
Anosmia	155 (13.2)	24.7
Taste	105 (9.0)	30.3
Coronavirus	69 (5.9)	36.4
Smell	52 (4.4)	44.8
Dysgeusia	47 (4.0)	41.5
Taste loss/loss of taste	47 (4.0)	26.6
Radiotherapy	26 (2.2)	55.4
Hyposmia	23 (2.0)	70.7
Children	22 (1.9)	5.1
Epidemiology	21 (1.8)	11.5
Symptoms	21 (1.8)	3.1
Head and neck cancer	20 (1.7)	34.3

**Table 3:** Diseases/treatments (other than COVID-19) associated with taste loss as revealed by ageusia research.

Disease/Treatment	Number of Publications (% of 1170)	Citations per Publication (CPP)
Radiotherapy	26 (2.2)	55.4
Head and neck cancer	20 (1.7)	34.3
Guillain-Barré syndrome	11 (0.9)	18.7
Chemotherapy	8 (0.7)	21.6
Parkinson’s disease	6 (0.5)	77.0
Hypertension	5 (0.4)	9.0
Obesity	5 (0.4)	5.4
Chronic rhinosinusitis	4 (0.3)	101.5
Multiple sclerosis	4 (0.3)	7.8
Vismodegib	4 (0.3)	47.3
Advanced basal cell carcinoma	3 (0.3)	26.7
Amyotrophic lateral sclerosis	3 (0.3)	2.7
Clopidogrel	3 (0.3)	1.3
Diabetes mellitus	3 (0.3)	20.7
Sjogren’s syndrome	3 (0.3)	6.7
Sonidegib	3 (0.3)	29.0

**Table 4:** Top 10 most cited ageusia research publications.

Paper	Reference	Number of Citations	Context of Citations (%, Support, Contrast, Mention)
Olfactory and gustatory dysfunctions as a clinical presentation of mild-to-moderate forms of the coronavirus disease (COVID-19): a multicenter European study	[32]	1194	6.6, 2.0, 91.4
Neurological associations of COVID-19	[33]	763	2.8, 0.4, 96.8
Smell and taste disorders, a study of 750 patients from the University of Pennsylvania Smell and Taste Center	[34]	639	4.0, 0.9, 95.1
Real-time tracking of self-reported symptoms to predict potential COVID-19	[35]	515	6.5, 1.0, 92.5
Oral sequelae of head and neck radiotherapy	[36]	511	2.5, 0.2, 97.3
Inhibiting the Hedgehog Pathway in Patients with the Basal-Cell Nevus Syndrome	[37]	387	1.7, 0.3, 98.0
Smell dysfunction: a biomarker for COVID-19	[38]	358	8.4, 0.8, 90.8
Miller Fisher syndrome and polyneuritis cranialis in COVID-19	[39]	355	2.3, 0.8, 96.9
Neuropathogenesis and Neurologic Manifestations of the Coronaviruses in the Age of Coronavirus Disease 2019: a review	[40]	350	0.5, 0, 99.5
Anosmia and Ageusia: Common Findings in COVID-19 Patients	[41]	350	3.8, 0.4, 95.8

The number of citations came from the Web of Science Core Collection whereas their context came from Scite. Since the two databases collected citations differently, they could not be considered interchangeably. Readers should refer to the Materials and Methods section for the exact sub-datasets and coverage span involved in this study.

## Data Availability

Data is available from the Web of Science (https://www.webofscience.com/, accessed on 7 March 2022) and Scite (https://scite.ai/, accessed on 7 March 2022).
